# Elements of Success

**Published:** 1893-08

**Authors:** C. W. Steele

**Affiliations:** Barre, Vt.


### Elements of Success.

BY C. W. STEELE, D.D.S., BARRE, VT.

Read before the Vermont State Dental Society, March, 1893.

True worth is always found in company with modesty, and self-satisfaction is a sure impediment to mental improvement.

The true elements of success will never be exemplified in dentistry until the common backbiting and trivial contentions among brother practitioners shall be laid aside. Any member of the dental profession may obtain great success in performing well all that the usual calls in practice demand of him.

Our dictionaries tell us that the dentist is one who makes it his business to extract and repair the natural teeth, also to insert artificial dentures. This definition might well apply if students were taught that these three branches were sufficient preparation for the dentist, but considering the skill and duties required of the modern dentist, we find this definition covers but a small portion of the ground.

At the present time the dentist (and more especially a country practitioner) is expected to follow all the requirements embraced in the profession, but I hope the time is not far distant when dentistry may be divided into specialties, and each branch requiring one who is thoroughly educated in the department of his special calling, be it mechanical, operating or surgical, for one thing pursued to its completeness is worth a dozen things left unfinished.

The highest aim of the physician is to prolong life, and the highest ambition of every dentist should be to preserve the natural teeth.

One more element of success and dental discipline is to be found in our dental colleges. Where would our profession have been to-day had it not been for the elevating factor of our dental colleges?

While so many are disposed to criticise them, yet an unprejudiced mind must admit that without them dentistry to-day would be largely a mechanical art, and its status would be far below what it now is.

To be sure the college education is but the framework of the house, and it remains for us by careful study, close observation and increasing skill to complete and adorn the edifice.

As a member of the profession, what does the fact of our holding a diploma from some reputable college signify? This is better answered by Edward Everett Hale, who says: "Every diploma given in a liberal profession contains three pledges, first a pledge to learn for all, second a pledge to practice for all, third a pledge to teach for all," and therefore it becomes a matter of wise discrimination as to what we say, how much we say, and when we say it.

And as regards the D.D.S. we cannot have a grander title, and it is he who fulfills to the highest degree, does credit to himself, his profession and his alma mater, that rises the highest and makes the best man.

The possession of knowledge and the power to apply it are very different; our greatest orators and most fluent debaters are by no means always the best or most successful practitioners.

Presupposing that you have been educated for the profession of dentistry, and that your diploma entitles you to be considered equipped in all points for its practice, there are many things which will determine your success or your failure; there must be enthusiasm and devotion; one must not be content with his knowledge, but be constantly increasing it, keep abreast with the times and learn the latest inventions and discoveries. To be sure we can not all be discoverers or revolutionizers like Edison, Koller or Goodyear, but if perchance we make a discovery either accidentally or from long study, let us bring it before the profession, bring it to the State Dental Meeting for criticism, and if there is any good in your discovery or invention you will find it out, and if there isn't, well, you will find it out just the same.

The question is often asked me in the early summer months, "Are you going to take a vacation? If so, when are you going, where are you going, and how long will you be gone?" Now, you must all agree with me when I say that dentistry is a trying occupation; it entails upon the individual who follows it faithfully an undue nervous strain which, if too long continued, results in some kind of collapse.

The height and depth of human accomplishments should not be measured by money. How can a man advisedly ignore nature to the extent of digging after the dollars and cents to the exclusion of everything else? It is an injustice to himself and family that he should jeopardize his health and temper by a blind policy of continuous application.

The question is often asked, "Does it pay for a dentist to follow, month in and month out, the routine of office work without permitting himself, at reasonable intervals, the relaxation and recuperation which a judiciously spent vacation affords?"

I am thoroughly convinced that the men who have accumulated the most money in dentistry are men who have taken frequent vacations, men who have traveled and enlarged their ideas in intercourse with other dentists and with the world.

A man becomes a better individual and a better dentist by extended experience, and the most observant man in the world can not gain a very extended experience in a dental office alone.

If I were asked what I considered the most essential element, and what was most conducive to success in the practice of dentistry, I would unhesitatingly answer judgment, and by judgment as applied to the dental practice, I would mean the faculty which enables us to pursue that course in practice, and to select the means and methods most suitable to our own individual surroundings, but, as judgment is progressive and experience one of the essential elements of mature judgment, it is not strange that errors often occur at this point; many a man has become mentally dwarfed by the very poverty of his judgment.

After a student has passed through the collegiate course and finds himself still alive, and more frightened than hurt, with his diploma safely lodged under his arm, the next important subject which he must consider is that of a location; and upon this we can not place too much emphasis, for failing in that, as immature judgment often does, a change should be made, and if made at all, as soon as possible, as a dentist's reputation constitutes a large part of his capital stock.

To be sure we meet with perplexities on every corner but the mental calibre of every dentist should be of such dimensions as to overcome such difficulties and as Father Atkinson once expressed it, " That perplexities are most frequent with the incompetent grumbler who sees the obstacles in his pathway but lacks judgment and perception of earnestness sufficient to overcome them."

One element very essential to a dental practitioner is that he must be a lineal descendant of Job, for in him patience must have its perfect work. He must be a judge of character and know how to influence his patients, for in dentistry as in everything else, granted that both have other advantages equal, it is the man with the most patience and judgment that succeeds.

In conclusion I would mention one other line of thought, and that is how to deal with another man's patients, and this I consider a matter of great importance.

Very few professional men have been so blessed as to have passed through even a limited career without being forced into unpleasantness, through misunderstanding arising out of their relation with other men's patients.

Professional ethics is not a trivial consideration but a matter of great importance, and when a patient comes to us after having had work done by another dentist we should be cautious in what we say. If there is fault, and we are asked the nature of that fault, we should modify our criticism as much as possible, and even then in the kindest spirit and mildest language.

Many are apt to condemn and criticise on slight evidence, but were these same critics held to account for their own failures their excuses would be many and their judgment obscure.

It is generally accepted by professional men as being quite outside the bounds of etiquette, to criticise unfavorably the work of another qualified practitioner, and the offense is still more aggravated when the criticism is offered without solicitation.

But if work has been well done make special emphasis of the fact. I believe in true courtesy and in being charitable to other dentists. Still we ought not to deceive our patients, and I fancy no better advice can be given as regards professional ethics than the motto, " Do to others as you would that they should do to you."

Much depends on the careful consideration of the code of ethics in dentistry. Measure yourself truthfully and critically, and relieve yourself, if possible, of the awkward position of being called an inveterate grumbler and chronic kicker, for the general principles of equity and common sense should be a sufficient guide to us in our relations with our patients, our profession and ourselves.

